# A Bio-Inspired Model-Based Approach for Context-Aware Post-WIMP Tele-Rehabilitation †

**DOI:** 10.3390/s16101689

**Published:** 2016-10-13

**Authors:** Víctor López-Jaquero, Arturo C. Rodríguez, Miguel A. Teruel, Francisco Montero, Elena Navarro, Pascual Gonzalez

**Affiliations:** Louise Research Group, Computing Systems Department, University of Castilla-La Mancha, Albacete 02071, Spain; victor@dsi.uclm.es (V.L.-J.); art.c.rodriguez@gmail.com (A.C.R.); MiguelAngel.Teruel@uclm.es (M.A.T.); fmontero@dsi.uclm.es (F.M.); pgonzalez@dsi.uclm.es (P.G.)

**Keywords:** tele-rehabilitation, context-aware, task model, presentation model, Post-WIMP systems

## Abstract

Tele-rehabilitation is one of the main domains where Information and Communication Technologies (ICT) have been proven useful to move healthcare from care centers to patients’ home. Moreover, patients, especially those carrying out a physical therapy, cannot use a traditional Window, Icon, Menu, Pointer (WIMP) system, but they need to interact in a natural way, that is, there is a need to move from WIMP systems to Post-WIMP ones. Moreover, tele-rehabilitation systems should be developed following the context-aware approach, so that they are able to adapt to the patients’ context to provide them with usable and effective therapies. In this work a model-based approach is presented to assist stakeholders in the development of context-aware Post-WIMP tele-rehabilitation systems. It entails three different models: (i) a task model for designing the rehabilitation tasks; (ii) a context model to facilitate the adaptation of these tasks to the context; and (iii) a bio-inspired presentation model to specify thoroughly how such tasks should be performed by the patients. Our proposal overcomes one of the limitations of the model-based approach for the development of context-aware systems supporting the specification of non-functional requirements. Finally, a case study is used to illustrate how this proposal can be put into practice to design a real world rehabilitation task.

## 1. Introduction

The use of Information and Communication Technologies (ICT) is becoming ubiquitous. They have been proven useful in many aspects of daily routines but, perhaps, healthcare is one the most relevant ones, because of the potential impact it can have in our quality of life. It has been claimed by many international organizations, such as the European Union [1], that ICT is the only feasible alternative to provide citizens with sustainable healthcare systems. The use of ICT has opened the door to different types of solutions in this domain. Tele-rehabilitation [2] is one of them as it helps to move healthcare from hospitals and care centres to patients’ home exploiting computing technologies, telecommunications, etc.

Among the different types of remote rehabilitation services [3] available nowadays, tele-therapy is the one that has a higher impact in terms of cost saving, as it focuses on delivering therapeutic activities to patients’ home. Several approaches have been followed in this context. For instance, different proposals have been presented that exploit, successfully, the use of Virtual Reality to develop Virtual Environments for motor rehabilitation [4], while others, [5,6], take advantage of different actuators to stimulate patients during their treatment. Other systems [7], exploit different sensors and devices, such as Microsoft Kinect, cameras, etc., to provide patients with cognitive rehabilitation. However, it is worth noting that tele-therapy does not mean unattended therapy, but just the opposite, therapy designed and monitored by specialists to prevent any damage or negative effect and take the most of the rehabilitation process. However, the fact that patient is at home encompasses several challenges and needs that must be properly addressed to develop good solutions. One of them is that tele-rehabilitation systems must provide specialists with facilities to define bespoke therapies, that is, therapies adapted to the specific needs of the patients. This encourages patients’ motivation as well as the achievement of better results. This has led us to develop a tool that enables therapists to design bespoke therapies which is part of Vi-SMARt, a system whose final aim is to provide support for physical [8] and cognitive rehabilitation [9]. These therapies, once designed, are executed, monitored and adapted thanks to a fuzzy inference system that has been integrated into Vi-SMARt [10].

Moreover, patients, especially those carrying out a physical therapy, cannot use a traditional Windows, Icon, Menu, Pointer (WIMP) system, but they need to move and interact in a natural way, so the system should be able to detect their movements, check their physical condition while they are doing their exercises, and inform them about their progress. That justifies why there is a clear need to switch from WIMP systems to Post-WIMP systems. Post-WIMP is a term coined to describe work about user interfaces that go beyond the paradigm of WIMP interfaces [11]. This new generation of interfaces has been fueled by both the advances in technology and an improved understanding of human psychology. They were defined by van Dam [11] as interfaces “containing at least one interaction technique not dependent on classical 2D widgets such as menus and icons”, being context aware computing one of such styles. According to Baldauf et al. [12] “context-aware systems are able to adapt their operations to the current context without explicit user intervention and thus aim at increasing usability and effectiveness by taking environmental context into account”. As can be noticed, tele-rehabilitation systems can be considered as context-aware systems, as they must be able to adapt their behavior to the patients’ context in order to provide them with usable and effective therapies.

Unfortunately, there is no consensus about what context is, how it should be specified, etc., but on the contrary there is a myriad of proposals to address these issues, as described in Section 3.2, each one having their strengths and weaknesses. This variety emerges because most of them have been defined with a specific domain in mind, in order to provide stakeholders with enough expressive power. This follows one of the principles identified in [13], which claims that while developing context-aware systems the types, capabilities, names and properties of all entities relevant to the system at hand must be modeled, and, therefore, limiting their possible domain of application. Following this approach is how this work has been defined. In this paper we present our proposal for developing context-aware Post-WIMP tele-rehabilitation systems by using: (i) a task model for designing the physical rehabilitation tasks; (ii) a context model to facilitate their adaptation to a changing context; and finally; (iii) a bio-inspired presentation model to customize how the physical rehabilitation tasks are offered to the patients.

This work has been structured as follows. After this introduction, related work is described in Section 2, identifying both current proposals for tasks and context-aware modeling. Then, in Section 3 the main ideas of this work are described, by explaining the bio-inspired model-based approach, reviewing the task model, the context-aware model and the presentation model that have been defined to support such ideas. How these models are put into practice is detailed in Section 4. Finally, the conclusions drawn and future work are presented in Section 5.

## 2. Related Work

Dey [14] defined a context-aware system as “a system that uses context to provide relevant information and services to the user where relevancy depends on the user’s task”. Moreover, in order to enable context-awareness, separating how context is acquired from how it is used is required [15]. The development of context-aware systems can be conducted following many approaches [16,17,18]. Among these techniques, those based on models introduce important advantages [18]. Model-based user interface development (Mb-UID) [19] and Model-Driven Engineering (MDE) [20] promote the systematic use of models in order to raise the level of abstraction at which user interfaces and software are specified, and to increase the level of automation in the development of user interfaces and software.

Mb-UID and MDE propose models to develop systems and models transformations to obtain target system’s software artifacts. However, there is a lack of consensus on the number and type of models that should be used to specify a system. Widely-used models [19]: (i) the *task model* that represents the activity functionality, identifying which tasks and interactions are needed to perform it; (ii) the *domain model*, which consists of any user visible, operable objects in the applications’ domain, representing the operational environment aspects; (iii) the *presentation model* shows, traditionally, visual elements needed for a WIMP (using windows, icons, menus and pointing devices) interaction; (iv) the *user model* is where the participants aspects are represented, characterizing the users and user groups; (v) *platform model* represents the physical infrastructure and the relationships between the devices involved; and (vi) the *environment model* includes spatio-temporal attributes, rules, and functions that characterize the physical and social places when/where the interaction will take place, or is actually taking place. The last three models—user, platform and environment—aim to characterize the context of use of a system.

Mb-UID approaches are useful techniques for the development of WIMP user interfaces [19] or graphical user interfaces (GUI). As Turk [21] stated “The main reason that GUIs became so popular is that they were introduced as application-independent platforms. Because of this, developers could build applications on top of a consistent event-based architecture, using a common toolkit of widgets with a consistent look and feel”. In this sense, WIMP user interfaces are visible to the user and for the users the system is its user interface.

However, post-WIMP interfaces rely on new interaction techniques such as 3D desktop graphics, multimodal interfaces, tangible interfaces and virtual or augmented reality. These new techniques arise from a need to support a more natural, flexible, efficient and powerfully expressive interaction easy to learn and use. In these post-WIMP interfaces, there is no standardized set of user interface widgets, but interaction is based, for instance, on gestures or movements and users can directly interact with his/her body. This type of interaction is especially useful for the rehabilitation domain. As recent studies suggest the use of motion tracking devices such as Kinect “carries the potential to become a future cornerstone of widely dispersed care and rehabilitation systems” [22]. The traditional WIMP user interface is now becoming invisible or transparent to the user and improving the attractiveness and intuitiveness of the rehabilitation therapies [23]. Thus, creating context-aware systems under post-WIMP paradigm is challenging.

In this paper, a model-based approach for the development of context-aware post-WIMP tele-rehabilitation system is discussed, and a set of models is presented to cope with the particularities of the development of such systems. The core models considered are the task and the context ones. These models are enriched with a presentation model to further refine the interaction design. Next, these core models—task and context—are reviewed to discuss if available model-based techniques can be used to support the specification and development of context-aware post-WIMP systems. Our intention is not to provide a new engineering process for context-aware systems, however, in this section, we aim at identifying shortcomings of the traditional techniques for task and context modeling in the development of this kind of systems.

### 2.1. Tasks Analysis and Models

**Figure 1:**
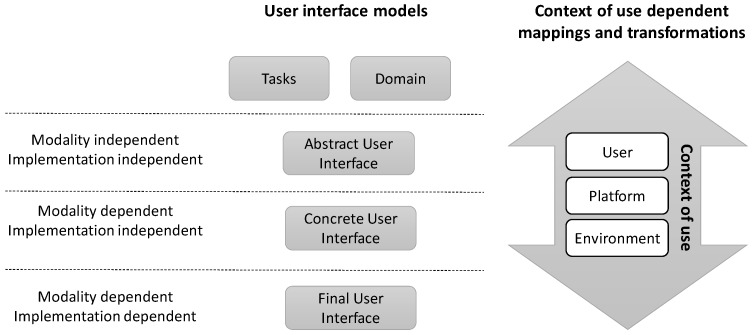
A simplified version of the Cameleon Reference Framework (CRF). Mappings and transformations between levels of abstraction depend on the context of use.

Task analysis is an activity that analyses what a user is required to do in terms of actions and/or cognitive processes to achieve a task. A detailed task analysis can be conducted to understand the current system and the information flows within. It is usually performed in the requirements elicitation phase during the user interface development process. Task analysis makes it possible to design and allocate tasks appropriately within the new system. Usually, the result of task analysis is the input for subsequent task modeling. Successful examples of task analysis can be found in model-based user interface development [19] (see Figure 1).

As depicted in Figure 1, model-based user interface development techniques make explicit a set of UI models (e.g., tasks, abstract UI, concrete UI, final UI and context of use) and their interrelationships (mappings), to serve as a common vocabulary within the HCI Engineering community to discuss and express different perspectives on a UI. Among these models, the task model corresponds to the hierarchies of tasks that need to be performed on/with domain objects (or domain concepts) in a specific temporal logical order for achieving users’ goals (during the interaction with the UI). Several notations are available to support task analysis and task modelling. Some relevant examples of these notations are: Hierarchical Task Analysis (HTA) [24], Collaborative Systems Requirements Modelling Language (CSRML) [25], ConcurTaskTrees (CTT) [26], Kernel of Model for Activity Description (K-MAD) [27] and Human-centered Assessment and Modeling to Support *Task* Engineering for Resilient Systems (HAMSTERS) [28]. However, there are few examples of usage of these notations in the development of post-WIMP user interfaces. Usually, the aforementioned notations have been used for developing WIMP user interfaces and only HTA, CSRML and K-MAD have been considered for post-WIMP user interfaces.

Hierarchical task analysis [24] (HTA) is a widely used type of task analysis where a high-level task is decomposed into a hierarchy of subtasks. An HTA model is sometimes referred to as a hierarchical decomposition. HTA is useful for decomposing complex tasks, but has a narrow view of the task, and normally is used in conjunction with other methods of task analysis to increase its effectiveness [29]. Task analysis can be also done using CSRML [25] (Collaborative Systems Requirements Modeling Language). It is an extension of i* [30] to deal with the specification of the requirements of collaborative systems in which collaboration and awareness of other users’ presence/actions are crucial. The concept of task in CSRML is the same as in i*. They only differ in the notation introduced to define the importance of a task, and also in the different types of tasks identified in CSRML. It has been extended also to support the development of post-WIMP user interfaces [31]. Since CSRML is a Goal-Oriented Requirement Engineering language, its task model can also benefit from its capabilities to specify goals (a certain state in the world that a stakeholder would like to achieve) and softgoals (a goal whose achieving conditions are not sharply detailed, usually related to non-functional requirements) [32]. Furthermore, the extension of CSRML used here includes also capabilities for both awareness-demanding tasks as well as relationships with the task execution context. The K-MAD task model is hierarchical. It represents the user’s activity as a tree of tasks, starting from the most general level (root) to the most detailed level (elementary actions). In order to express activities, the K-MAD [27] model consists of components and scheduling characteristics and it has been used in the development of user interfaces of mixed interactive systems (MIS) [33].

ConcurTaskTrees [26,34] (CTT) is a notation and a tool (CTTE) for task model specification widely used in the design of interactive applications specifically tailored for WIMP model-based user interface design. This notation is focused on activities. Thus, it enables designers to focus on the most relevant aspects when designing interactive applications that encompasses both user and system-related aspects. It also avoids low level implementation details, which at the design stage would only obscure the decisions to make. In the same way, HAMSTERS [28] (Human-centered Assessment and Modeling to Support Task Engineering for Resilient Systems) is a task modeling language with a supporting tool. It is highly inspired by existing notations and tools (e.g., CTT and CTTE) and it is useful to describe and analyze the impact of system faults and human errors in an integrated manner. These last proposals are modality and platform independent, however these task model proposals are context-dependent, that is, the UI designer has in mind, in an explicit or implicit manner, the context of use (specially the platform) and the interaction between the user and the machine. All these task models share some features that should be taken into account: the tasks are defined in terms of goals and operations, each abstract task is divided into subtasks defining a hierarchy and finally there should be a set of rules that control the execution of subtasks.

Some models usually used along with the task model are the user interface presentation model and the context model. The user interface presentation model expresses the UI in terms of interaction elements, as well as the relationships among them. The presentation model is sometimes decomposed into abstract and concrete (see Figure 1). The interaction elements included in the abstract presentation model are independent of any implementation technology as well as any modality (e.g., graphical, vocal, gestural, haptic and 3D). On the other hand, the ones included in the concrete presentation are platform independent, but dependent on modality. In other approaches, a single presentation model is often used omitting the abstract presentation model. Lastly, another extra model is considered to represent the actual application the user interacts with. It is the so called final user interface (see Figure 1). This final user interface can be generated from the presentation and related models in model-driven approaches.

In model-based user interface proposals, the term *context of use* and the context model denote an information space structured into three main models: user, platform, and environment. The adaptation of the user interface depends often on both the context model and the interaction between the user and the system [20,35]. In this sense, information from sensors can be relevant to characterize the context and to properly adapt an interactive system. For instance, knowing the stress or fatigue level of users can be relevant to adapt the therapies in a rehabilitation system. Moreover, the application domain, for instance, can provide extra relevant information that completes the characteristics of the context of use. For example, in a rehabilitation environment, knowing that the interactive system supports physical rehabilitation activities provides useful information.

Regarding the task model and how it can be related to a specific context, Pribeau et al. [36] discussed three different approaches to extend task models by including context features: monolithic, graph oriented and context-sensitive separation. The first approach includes a global task model that directly considers context-insensitive and context-sensitive parts together. The second one extracts sub-trees resulting from the context-sensitive part into separate trees, by separating context-sensitive and non-context sensitive parts of a task model. The last approach includes three types of trees: context-insensitive, context-sensitive and decision. This last type of tree enables defining all contextual conditions related to the potential context situations. In our approach we define two task models: a context-insensitive task model and a context-sensitive one associated to each potential context.

In this paper, task, presentation and context models are used together in order to support the development of physical tele-rehabilitation Post-WIMP systems. 

### 2.2. Context Models

As aforementioned, post-WIMP systems require context information and, therefore, context modeling is an activity to be considered in their development. Context modeling has been widely explored over the last years [37], although it is within the domain of context-aware system where has had a further interest in its study [18,38,39]. Since context-aware applications and ubiquitous computing are becoming increasingly widespread, it is necessary to develop languages expressive enough to specify context elements in order to improve their development process [40]. There are numerous approaches to describe the context of a system but, in spite of considering some common concepts like Environment, User and Platform (or physical world, individuals and technology as Bauer stated [39]), the way they interpret them and how they are modeled are very heterogeneous.

The concept of context itself can even differ depending on the proposal. For instance, early definitions of context in the literature related context to location and the ability to detect nearby devices [41]. Rodden et al. [42] extended this definition by considering that the context entails five different dimensions: Infrastructure context, Application context, System context, Location context and Physical context. However, during the last years, the concept of context has evolved. For Vieira et al. [43], the context is “a dynamic concept that has a strong relation with an agent’s focus of attention”. On the other hand, Dey [14] defines context as “any information that can be used to characterize the situation of an entity. An entity is a person, a place or object that is considered relevant to the interaction between a user and an application, including the user and application themselves”. Soylu et al. [44] describe Dey’s definition as generic and open-ended, covering the context as a whole. A more recent definition stated by Knappmeyer et al. [40] extends the concept of entity proposed by Dey and establishes different context information categories. They define context as “any information that provides knowledge and characteristics about an entity (a user, an application/service, a device, or a spatially bound smart place) which is relevant for the interaction between the entities themselves and with the digital world. Context can be categorized as being static, dynamic and rapidly changing”. Thus, for these authors, it is not possible to predefine all the dimensions that conform the context, defining it as “an open concept since it is not limited with one’s imagination”. However, Dey’s definition of context has been widely accepted by many authors, such as Vale and Hammoudi [45]. They only highlight that this diversity regarding the meaning of context makes context-aware programming such a challenging and complex task.

This variety of context interpretations clearly hinders an appropriate definition of a language for context modeling. In most cases, the approach followed is driven by the type of application to be developed. Context description is mostly used in context-aware applications, but such type of applications is not the only one that requires a detailed context description. For instance, in a model-driven development of user interfaces, a suitable context specification may be also necessary, as UsiXML shows [46], one of the foundations of our proposal. UsiXML is a framework for describing user interfaces by using different abstraction levels. Thus, each abstraction level represents a view of the interface that is being modeled. For this purpose, UsiXML offers a set of meta-models, being the context meta-model one of them. This meta-model is used to specify the information of entities that affect a system and this information is mainly used to achieve a correct adaptation to different context of use. It focuses on the characteristics of such entities (internal point of view) and on the interaction zones (external point of view). Moreover, the internal point of view is divided into two abstraction levels. The high abstraction level representation enables designer to specify the characteristics and attributes of the entities and the low abstraction level enables them to establish constraints on such attributes. This context meta-model offers a generic tool to describe the context of user interfaces, however is not expressive enough to model *dynamic context information*, i.e., the context information that changes at run-time and is relevant to the system behavior. This dynamic information is necessary for designing Post-WIMP interfaces in a suitable way, given that this information can be used to improve the system execution. Moreover, in these kinds of systems, the interaction could depend on that dynamic context information, such as the heart rate of the user or the position of her/his limbs.

Regarding context-aware modelling, Vale and Hammoudi [45] propose both a context meta-model and a model-driven development (MDD) process for context-aware development. This meta-model is based on ontological concepts and describe context using two abstraction levels that match the M2 and M1 layers of model-driven architecture (MDA). The context information in M1 is limited to four concepts: Time, Location, Device and Profile. However, this context description is, perhaps, too limited and omits a detailed description of the user or environment as well as about the relations between dynamic information and the devices in charge of measuring it, as Schmidt already stated in [47]. When designing context-aware applications not only information about the context is relevant, but also about the devices or sensors that monitor such information. In this sense, Schimdt’s proposal focuses on implicit Human-Computer Interaction (iHCI) and offers an XML-based language for specifying context taking into account sensors, contextual variables and system reactions. However, the approach lacks of expressive power to describe structural context information and has not been included into a model-driven development process. Sigg et al. [48] go one step further and propose a method to predict context. This method, called *Alignment Prediction Approach*, relies on typical context patterns and can handle multidimensional and multi-type context sequences. Sigg et al. divide the context in six dimensions: Identity, Location, Time, Environment, Constitution and Activity and each dimension gathers different types of context information. However, this proposal is not aimed to specify the context at design time, but to provide just a description for context prediction. Finally, using a MDD approach Hoyos et al. [40] present a proposal that includes six different types of context information (physical, environment, computational, personal, social and task). Moreover, they include the definition of a domain-specific language to describe this context information and they include three modifiers to specify three features (quality, historic and static). Although in their proposal they include several relevant views of context information, and take into account the task as one of them, as other MDD approaches, they do not include any information about other non-functional requirements [18] that should be analysed in post-WIMP system development.

Although the great majority of proposals have been defined for context-aware systems, there are a few ones that associate context model specifically to Post-WIMP applications. In [49] a context model for designing interactive tabletop applications, combining both tangible and virtual objects is described. It takes into account three views: user, platform, and environment. Each view has its own meta-model which is adapted to these specific applications. Although this proposal analyses the same three views of the context as ours, the relationship between the tasks where the context elements are required and other elements of the context model are less general and they are adapted to the particularities of the design of interactive tabletop applications that include both virtual and tangible objects.

To sum up, a context model must consider not only dynamic and static context information that take into account all the features related to context information [39], but also all the potential ways of interacting with the system (interaction modalities) and other non-functional requirements. Choosing the right interaction modality will depend on the user model and the environment model, as well as on the devices that comprise the Post-WIMP system at hand. Modelling the interaction modalities of a system enables designers to contextualize other models, such as the Abstract User Interface Model or Task Model, so that, systematic transformations can be applied to generate models at a lower level of abstraction as, for instance, the Concrete User Interface Model. On the other hand, the inclusion of softgoals and awareness information enables designers to describe some non-functional requirements needed in the development of Post-WIMP systems and to establish the relationship of this information with other elements of the context.

## 3. A Model-Based Approach for Context-Aware Post-WIMP Tele-Rehabilitation

When following a model-based approach for design, different models can be included in the design process. In this work three models are used together to foster the design of Post-WIMP tele-rehabilitation systems under the umbrella of model-based design. The models used are the task, the context and the presentation models. The task model enables the specification of what tasks the rehabilitation activity includes. This task model is enriched with the context model to describe all those features of the surrounding context of use of the patient that can provide meaningful information to improve the tele-rehabilitation. Lastly, the presentation model details how each step of the rehabilitation activity should be done. In the next sections, these three models are explained in depth.

### 3.1. A Task Model for Post-WIMP Tele-Rehabilitation

This section presents the bio-inspired task model devised. More specifically, the task model that is described in this section is an extension of CSRML supporting the specification of Post-WIMP tasks by adding the following features:

- Context elements: each CSRML task can be enriched with context information details including whether or not each task is for physical rehabilitation as well as the context elements to be monitored, because of their relevance to the task.
- Awareness representation: this extension of CSRML adds awareness representation features in order to specify the context elements that a user performing a task must be aware of.
- CTT compatibility: temporal restrictions and systems tasks were added in order to ensure the compatibility of this extension of CSRML with CTT making it easier for the practitioners of CTT to embrace CSRML.

It is worth noting that, since CSRML is a Goal-Oriented proposal [50], its tasks model can represents goals and softgoals, thus enabling the representation of systems status to achieve (goals) and quality requirements whose achievement is not clearly defined (softgoals). For its definition, the CSRML [25] task model has been adapted to enable the specification of bio-inspired tasks. CSRML has been selected because it already supports the requirements specification of collaborative features, one of the main cornerstones of Post-WIMP systems. However, in order to make it suitable for the specification of bio-inspired tasks, it has been modified to provide expressiveness for contextualized and physical rehabilitation tasks. Therefore, by using this model, not only classical user tasks can be specified, but also Post-WIMP tasks to specify that users will interact with the system by using their body movements. In addition, by contextualizing the user tasks they may be performed or monitored in a different manner, depending on the context (e.g., user abilities, available hardware, lighting conditions, etc.). Moreover, the relationship among tasks and contextual elements is specified by means of the context model that is explained in Section 3.2.

**Figure 2:**
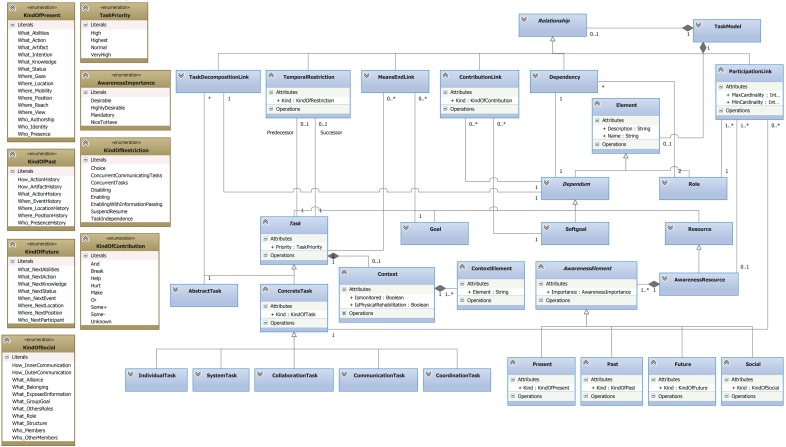
Bio-inspired task meta-model.

The underlying meta-model for specifying bio-inspired task models is illustrated in Figure 2. 

**Figure 3:**
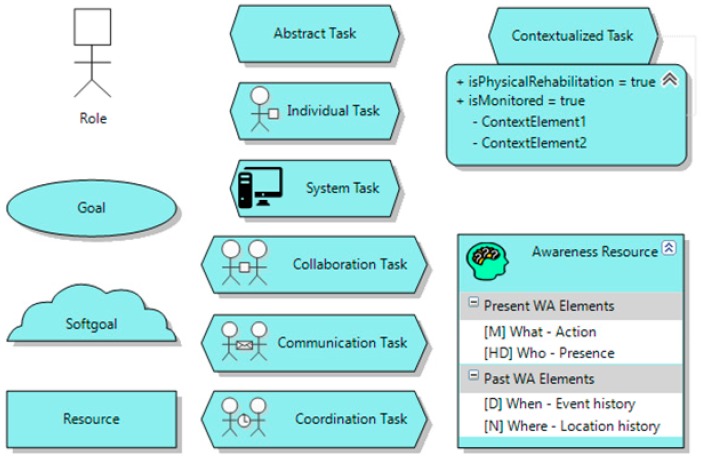
Elements of the bio-inspired task model.

In the following, a brief explanation of each one of the elements (see Figure 3 for their graphical description) of this meta-model is presented:

- *Role*: It is a designator for a set of related tasks to be carried out. An actor playing a role can participate in individual or collaborative tasks (through *participation links*) and it can be responsible for the achievement of a goal (through *responsibility links*).
- *Goal*: It answers “why?” questions. It describes a certain state of the world that an actor would like to achieve. However, a goal does no prescribe how it should be achieved.
- *Softgoal*: It is a condition in the world that an actor would like to achieve but, unlike the concept of (hard) goal, the condition for its achievement is not sharply defined. A softgoal is typically a quality attribute that constrains another element, such as a goal, task or resource.
- *Task*: It specifies a particular way of doing something. As can be seen in the meta-model (see Figure 2), this element has an importance level that corresponds to the development priority a task must have. In addition, two types of tasks have been identified:
  ■ *Abstract task*: it is an abstraction of a set of concrete tasks and, optionally, other elements.
  ■ *Concrete task*: These tasks are refinements of abstract tasks and they have roles responsible for their accomplishment. There are four types of concrete tasks: an *Individual task* is a task that an actor can perform without any kind of interaction with other actors. *Collaboration/Communication/Coordination tasks* require two or more actors to be involved in order to perform any kind of collaboration/communication/coordination between them. *System task* is a task that is carried out by the system or a non-human agent in an autonomous way (e.g., collect data of the environment or make a database backup).

Moreover, any kind of task can be contextualized. For this aim, as Figure 2 shows, a relationship has been defined between Task and Context. Figure 2 depicts how it will be graphically illustrated the relationship between a contextualized task and a context, to indicate whether the task can be monitored by which context elements (see Figure 3).

- *Context:* This element has been introduced to describe that a task can be contextualized. As can be observed, it has two attributes. *isMonitored* is used to indicate whether this task will be monitored by the system, and which *ContextualElements* will be in charge of carrying out such mission. *isPhysicalRehabilitation* is used to specify that a task is for physical rehabilitation. This attribute also indicates that the related task will be detailed in the Presentation Model, as described in Section 3.3.
- *Resource:* It is an entity (physical or informational) that the actor needs to achieve a goal or perform a task. The main concern about a resource is whether it is available and from whom.
- *Awareness Resource*: This element represents some awareness perceptions that a role needs to accomplish a task. As the meta-model shows (Figure 2), this element is used to identify which *Awareness Elements* can be of interest for the system at hand. All these elements have been identified by conducting a Thematic Synthesis of the existing awareness interpretations [25]. In addition, these elements are specialized according to their temporal category and classified according to their *importance* (*nice to have, desirable, highly desirable or mandatory*).

**Figure 4:**
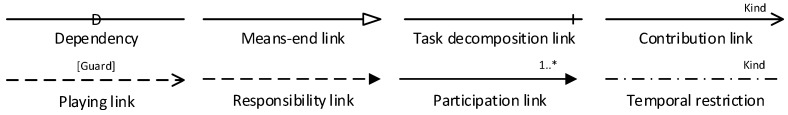
Relationships of the bio-inspired task model.

Now, once the bio-inspired elements have been defined, they can be related among them by means of the following set of relationships (Figure 4):

- *Dependency*: It is a relationship between a *depender* and a *dependee* for a *dependum*. The *depender* and the *dependee* are actors and the *dependum* can be a goal, a task, a resource, or a softgoal. The *depender* depends on the *dependee* for achieving a goal, performing a task, or using a resource. If the *dependee* fails to provide the *depender* with the required *dependum*, it becomes difficult or impossible for the *depender* to achieve the goal, perform the task, or use the resource. Based on the type of *dependum*, there are four types of dependencies: goal dependency, task dependency, resource dependency and softgoal dependency.
- *Means-end link*: It documents which softgoals, tasks, and/or resources contribute to achieve a goal. A means-end link also facilitates the documentation and evaluation of alternative ways to satisfy a goal [51], i.e., different decompositions of a goal into subgoals, tasks, and resources.
- *Task decomposition link*: A task decomposition link describes the essential elements of a task. A task decomposition link relates the task to its components, which can be any combination of sub-goals, sub-tasks, resources, and softgoals. The decomposition of a task can thus comprise sub-tasks that must be performed, sub-goals that must be achieved, resources that are needed, and softgoals that typically define quality goals for the task.
- *Contribution link*: A contribution link documents an influence from a task or softgoal to other softgoal. Based on i*’s contributions [52], it is defined by means of the *kind* attribute of the corresponding meta-model element, and can be:
  ■ *Make*: A positive contribution strong enough to satisfice a softgoal.
  ■ *Some+*: A positive contribution whose strength is unknown.
  ■ *Help*: A partial positive contribution, not sufficient by itself to satisfice the softgoal.
  ■ *Unknown*: A contribution to a softgoal whose polarity is unknown.
  ■ *Some−*: A negative contribution whose strength is unknown.
  ■ *Hurt*: A partial negative contribution, not sufficient by itself to deny the softgoal.
  ■ *Or*: The parent is satisfied if any of the offspring are satisfied.
  ■ *And*: The parent is satisfied if all of the offspring are satisfied.
- *Playing link*: A playing link is used to represent when an actor plays a role. This link has a *guard* condition attribute that represents when a role can be played by an actor.
- Responsibility link*: It assigns a role (played by an actor) to a (soft) goal or task. This link represents who is the stakeholder responsible for a goal/task accomplishment.
- *Participation link*: It denotes who is involved in a task. This link has an attribute to specify its *cardinality*, i.e., the number of users that can be involved in a task. It can optionally have an *awareness resource* attached to it. In this way, it represents that the role has a special perception need (specified by means of the awareness resource) in order to participate in the task. Without this perception, the accomplishment of the task could be negatively affected or even the role could not be able to participate in the task.
- *Temporal restriction*: Based on CTT’s relationships [53], it represents a temporal restriction between two tasks. These temporal restrictions can be:
  ■ *Enabling* (>>): It specifies that the second task cannot begin until the first task is performed.
  ■ *Choice* ([]): It specifies that two tasks cannot be simultaneously enabled, so once one has started the other one is no longer enabled.
  ■ *Enabling with information passing* ([]>): It specifies the second task cannot be performed until the first task is performed, and that the information produced by the first task is used as input for the second one.
  ■ *Concurrent tasks* (|||): Tasks can be performed in any order, or at same time, including the possibility of starting a task before the other one has been completed.
  ■ *Concurrent communicating* tasks (|[]|): Tasks can exchange information while performed concurrently.
  ■ *Task independence* (|=|): Tasks can be performed in any order, but when one starts then it has to finish before the other one can start.
  ■ *Disabling* ([>): The first task (usually an iterative task) is completely interrupted by the second task.
  ■ *Suspend-Resume* (|>): First task can be interrupted by the second one. When the second terminates then the first one can be reactivated from the state reached before.

### 3.2. A Context Model for Post-WIMP Tele-Rehabilitation

**Figure 5:**
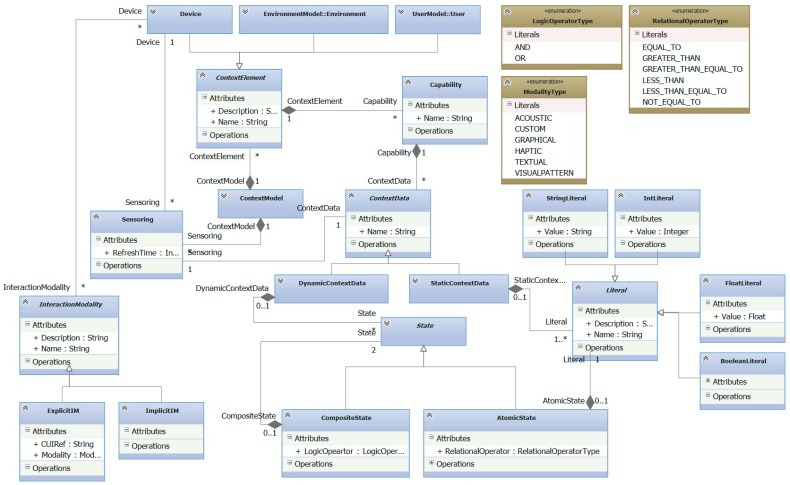
Context-meta-model.

In order to specify the context of a particular system, addressing the shortcomings detected in the aforementioned approaches, a new context meta-model, shown in Figure 5, has been defined. It has expressive power to describe the context at three levels. Firstly, it enables analysts to specify static features of context elements, i.e., those characteristics that will not change over time, as for instance physical features of the environment. These context elements can be either devices or the environment. Users could be also elements of context model but, due to the complexity of this concept a separate model is more suitable. Secondly, it also provides some mechanisms to specify dynamic features of these context elements, i.e., those features whose values change at run-time and are measured by the system. Note that the change of such values may affect the system behavior in some way, acting as conditions that may cause system reactions. Lastly, our context model aims at specifying the interaction modalities of the devices that compose the system. Next, the meta-classes of the meta-model are explained:

- *ContextModel*. It represents an instance of the context model.
- *ContextElement*. It represents an element whose description is relevant for the system. It can be a Device or the Environment (see below).
- *Device*. It is used to specify elements that belong to the system and whose description is relevant for context analysis. For instance, a Heart Rate Sensor, a Display, Leap Motion, or MS Kinect.
- *Environment*. Any physical element that is not part of the system, but affects the interaction process. For instance, the room where the system has been deployed.
- *Capability*. It is used to specify a set of features related to a context element. For instance, GPS capability of a mobile Device or ambient conditions of an Environment. The features that a *Capability* defines are called *ContextData*, and can be either dynamic, i.e., they change at run-time (*DynamicContextData*) or static (*StaticContextData*).
- *Literal*. It is used to define expressions that entail type, value and units of measurement of context data, e.g., IntLiteral: 180 bpm.
- *StaticContextData*. This is used to describe a feature of an element whose value either does not change at run-time, or its change is not relevant to the system. For instance, it could be relevant to a system to describe the visual accuracy of a user as part of the senses *Capability* as users with visual impairments will not interact with the system in the same way as users without visual impairments do. The features that represent the size of a room and the maximum resolution of a display are other examples of static information of the context. A *StaticContextData* element can also be related to one or more *Literals*, as some features only need one literal to be described, e.g., Frequency: 1.2 GHz, while others need two or more Literals, e.g., MaxResolution: 1900 px, 1080 px.
- *DynamicContextData*. It is used to represent features of the context that change at run-time affecting the system. For instance, the value of the feature Heart Rate of the *Capability* Vital Signs of a concrete *User*, should change over time as the heartbeat of the user carries out his tele-rehabilitation tasks. Unlike static context data, dynamic information is not directly related to Literals, but to *Conditions*.
- *State*. This is used to specify a value, range of values or state of dynamic data. When the current value (at run-time) of a dynamic context data matches a condition, i.e., it matches a value, range of values or a particular state, the system behavior is affected at run-time. This concept is similar to the Input Range defined by Schmidt [4].
- *InteractionModality*. This is used to represent the different types of interaction modalities. It is specialized in *ExplicitIM*, that represents those interactions that users carry out voluntarily and *ImplicitIM* that represents interaction that users carry out unintentionally.

Several types of explicit interaction modalities are considered:

- *Graphical*. It is used to specify the interaction by means of widgets that have graphical representation on the user interface. For instance, buttons, menus or combo boxes.
- *Acoustic*. It is employed to describe the interaction that has no graphical representation on the user interface, but enable users to interact with the system by means of sound. For instance, a voice recognition widget.
- *Haptic*. It is used to specify interactions that have no graphical representation on the user interface, but enable users to interact with the system by means of haptic stimulation, e.g., vibratory stimuli.
- *VisualPattern*. It is used to describe interactions with no graphical representation on the user interface, but enables users to interact with the system by means of gestures, changes of location or visual patterns. For instance, a TagVisualizer defined for MS PixelSense.
- *Custom*. It is employed to describe interactions that have no graphical representation and cannot be included in the previous interaction unit categories.

It is worth noting that the static description of the system enables analysts to document the basic conditions that surround the system, as well as the features of the supporting platform. These static features are represented by instances of *StaticContextData*. The analysis of this information facilitates the design of the user interface, i.e., the part of the system the user interacts with in an intentional way. In order to model the static part of the context, the basic context elements must be identified (users, devices and environments) by specifying its static features. Modeling both user and environment is very important in this process since the type of user and the features of the environment will determine which the best type of interaction is. It will also be a good starting point to choose the most suitable devices, taking into account their interaction modalities. This facilitates that different alternatives can be considered when the system is being defined. For instance, a user with vision problems could not be able to interact with the system through Graphical interaction modalities. In this case, acoustic interaction modalities, based on voice recognition widgets, would be a better choice. However, if the environment is noisy, a gesture based interaction would be chosen, instead of an acoustic one. Both environment and user context elements are out of the scope of this paper, since given their complexity they would make understanding the main focus of this paper harder.

The dynamic features of the context elements that change at run-time and whose changes are relevant for the system are represented as instances of *DynamicContextData*. The specification of these features enables analysts to document, not only basic attributes, but also the sensors to measure them. *Sensoring* establishes a relation between the *DynamicContextData* instance and the *Device* which represents the sensor that captures this dynamic information. Moreover, dynamic data may be related to a set of *States*. *State* describes those states of context data that are relevant to the system at run-time. These states may be referenced by other models so that system reactions could be related to specific conditions.

### 3.3. A Human-Movement-Inspired Presentation Model

Task models are considered as models that support the analysis and design of interactive systems, including context-aware ones. Nevertheless, some extra models are required, apart from the models of the surrounding context, to be able to render the tasks for a specific context of use so the users can interact with the system. Traditionally in the model-based user interface approach [53,54] a presentation model has served as the guide to specify how tasks will be presented, that is, to describe what interaction modalities and widgets for those modalities should be used. For instance, to perform a task to select the genre of a person, the designer could decide to use the graphical modality and then choose some widgets for that modality, such as a dropdown list or a group of check boxes, depending on the available space in the screen. On the contrary, the designer could decide to use the auditory modality so the user can choose the genre by using his voice.

In this work, we go one step further by considering that the interaction will be achieved by using post-WIMP technology aimed at supporting the detection of the movements required for a rehabilitation activity. The modality that will be used in our approach relies on the gestural modality, but in this case using a specific set of human movements. Thus, in our case instead of having widgets such as buttons, dropdown lists or check boxes, the widgets are the gestures derived from the many different movements a human can make. Therefore, now when the specialist describes in the task model that the patient should make a step of an activity, then the specialist will be able to choose from the presentation model what movements exactly the patient is supposed to perform to carry out such step. For example, the specialist in the task model can specify that the user should raise the arm, and then in the presentation model can specify precisely the starting and final position of the relevant joints involved in the task. This expressive power is aimed at supporting the prescription of proper and accurate therapies, rather than just text-based descriptions of the rehabilitation activities. 

By supporting the separation of concerns between the tasks and its presentation another extra feature can be achieved. A specific therapy created by the specialist can be customized to the context of use, and especially to the user, to foster the execution of adapted therapies rather than fixed ones. This can be achieved by choosing different presentations for the same task of the rehabilitation activity based on their suitability for the target context of use.

**Figure 6:**
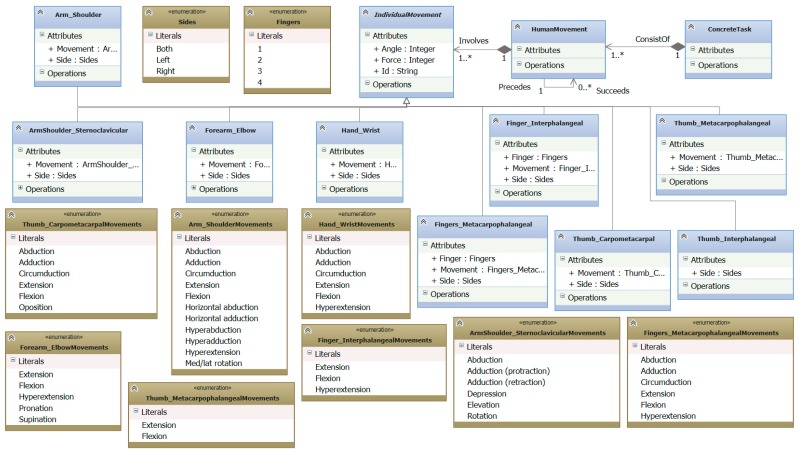
An excerpt of the bio-inspired presentation meta-model.

As aforementioned, our presentation meta-model describes the different movements a human can make, with the aim of describing precisely the expected movements a patient should do to properly perform the rehabilitation task. Figure 6 depicts an excerpt of this presentation meta-model. For the sake of clarity, in the figure only those elements of the meta-model related to the arm (that is the limb used in the case of study in this paper) are shown. Thus, the root of the meta-model is *ConcreteTask*. It is a task performed by a person by moving his body that can be monitored by a sensor. Please, note that only those tasks having *isPhysicalRehabilitation* attribute set to true can be related to our presentation model. i.e., only those tasks whose presentation is a body movement. Each *ConcreteTask* is composed of a set *HumanMovements*, which represents the movements a human will do to carry out the task. The relationships *Precedes and Succeeds* enable the specification of the ordering of these movements, when relevant. When a human makes a movement, a set of joints are set in motion. Therefore, a human movement involves moving some joints. Actually, the movements are described in terms of the segments defined by two joints. Each movement of a segment is an *IndividualMovement* in our model. The name of the *IndividualMovement* in the model has the format *<name_of_segment>_<name_of_source_joint>*. For instance, focusing on the arm movements, and starting from the closest segment to the trunk, these segments can be *ArmShoulder_Sternoclavicular*, *Arm_Shoulder*, *Forearm_Elbow* and *Hand_Wrist* (excluding all the segments in the fingers). Some of these segments also include the attribute *Side* to specify to what side of the body the segment belongs to. For instance, *Forearm_Elbow* could be from the left or the right arm. Moreover, depending on the segment, the set of possible movements it can do is constrained. Therefore, each segment includes an attribute *Movement*, where the specialist can specify exactly what movement of the segment the *IndividualMovement* requires. In the lower part of Figure 6 the enumerations describing what movements each segment can make is depicted. For example, the segment *Forearm_Elbow* can make an *Extension*, a *Flexion*, a *Hypertension*, a *Pronation* or a *Supination*. Lastly, the meta-model supports the specification of the angle of the movement and the required force to achieve it. The angle is expressed in degrees, according to the standard anti-clockwise notation, while the force is expressed in Newtons (N), or in Newton Meter (Nm) if it is a torque (rotation) force. These two features can be specified for each *IndividualMovement*. Please, see Section 4 for a detailed example of a specification of an *IndividualMovement*.

It is important to stand out that although the specification of movements can be tedious, it is greatly simplified when using a human virtual avatar so that specialists can describe the movements by manipulating the avatar or by using motion capturing devices [10,55]. With this approach we aim at capturing the movements of the specialist doing the movements the patient is supposed to do for his tele-rehabilitation. Then, the motion captured, either by using the avatar or the motion capturing system, is translated into the elements of the Presentation Model. 

How this meta-model, and the previously described context and task meta-models, can be used together to specify a therapy is illustrated in the next section.

## 4. Case Study

To better illustrate how all these models fit together a case study focused on the rehabilitation of upper limbs will be used in the following. This example is taken from [56], where different rehabilitation activities for elder people are described. In the activity chosen, the patient is asked to move her arms until she reaches a specific position to perform the so called *Upper Limb Rehabilitation* (ULR). This activity is aimed at strengthening the arms of the patient, therefore she is asked to perform the movements a number of repetitions (between 3 and 8 times). This activity is performed in an environment where a Microsoft Kinect is used to monitor the motion and positioning of the arms of the patient [57]. Some extra context information is considered also to improve the accuracy of the activity, including the heart rate, the skin conductance of the patient and the ambient light and noise levels of the room where the activity takes place. The main criteria for the selection of what sensors to use has relied on their availability and affordability (around 300€ the whole set), and also on previous experiences reported in the rehabilitation domain using those sensors [22]. All these context information provides the system with valuable information to adapt more accurately the activity to the patient. One of the parameters adapted to the patient is the accuracy of the position of the arms required to be considered successful, since not every person can achieve the same accuracy, especially the first time the activity is performed.

**Figure 7:**
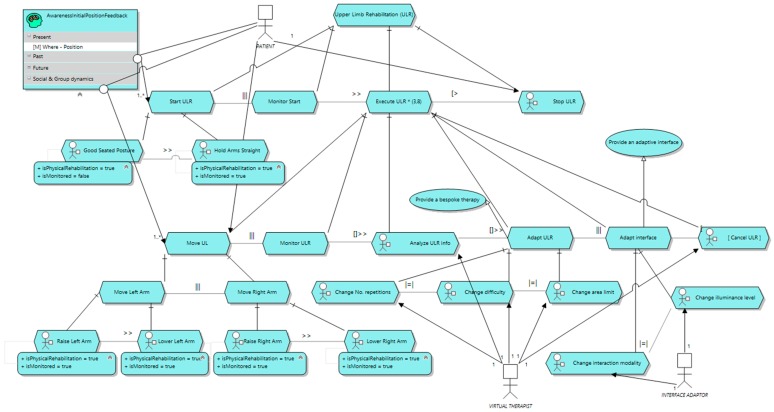
The upper limb rehabilitation core task model.

In Figure 7, the task model specified for the rehabilitation task aforementioned is depicted. No context information is included in this version of the model (task context-insensitive model). As can be observed, the model describes what tasks the rehabilitation exercise prescribes, together with some extra tasks that contribute to provide a better user experience. Furthermore, the temporal relationships (e.g., what task must be performed before another one) are also included by using the temporal restrictions enumerated in Section 3.1. The patient is responsible for starting the rehabilitation activity (*Start ULR*) to enable the execution and monitoring tasks (*Execute ULR*). First, the patient must adopt the starting position described (good seating position with arms held straight). The system is monitoring the initial situation of the patient (*Monitor Start*) to get some starting parameters, such as the current heart rate and the skin conductance level. Once the patient is ready, the execution of the rehabilitation starts. During the exercise the patient moves her upper limbs up to a position defined in the initial configuration of the rehabilitation activity (*Move UL*) from the starting position and back, and, at the same time, the system is monitoring a set of context variables (*Monitor ULR*). All the information gathered is analyzed by the system (*Analyze ULR info*) to make some decisions to adapt the configuration of the rehabilitation (*Change No. repetitions*, *Change difficulty* and *Change area limit*) and the environment (*Change interaction modality* and *Change illuminance level*). Both the system and the patient are able to stop the rehabilitation activity at any time (*Cancel ULR* and *Stop ULR*, respectively). Please note also how two awareness resources are included. These resources are aimed at providing the user with relevant information that contribute to help her in performing the tasks properly. In this case, the awareness resource provides her with her current position (i.e., the position of the user in the present). This is mandatory, since without feedback about the current position of her limbs the patient will not be able to know when she has reached the target position.

**Figure 8:**
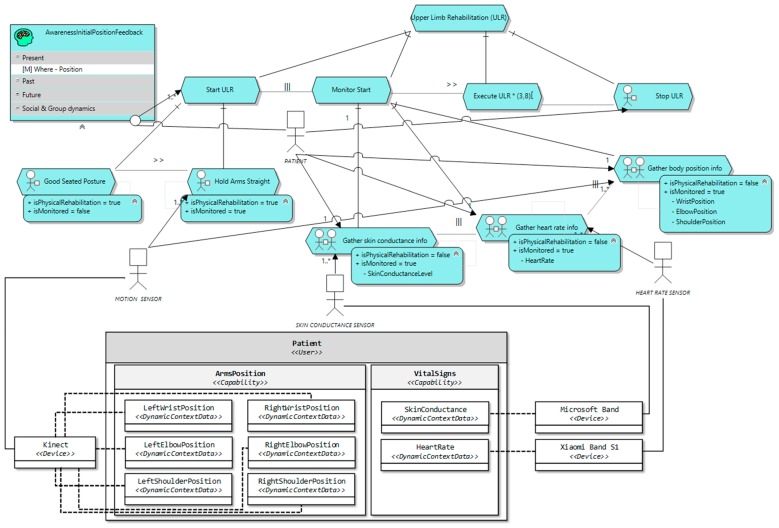
The upper limb rehabilitation task model with context information.

This task model can be further detailed by enriching the tasks with the surrounding context information relevant for their execution (task context-sensitive model). Thus, in Figure 8 a version of the task model including the context information is included. For the sake of clarity, only those context elements relevant to *Monitor Start* have been included. The specification of the context information for *Monitor ULR* would be similar.

The context elements relevant are specified as context resources for the corresponding task. e.g., *Gather skin conductance info* requires *SkinConductanceLevel*, as depicted in Figure 8. The purpose of the information from the context gathered in *Monitor Start* is two-folded: on the one hand, it enables checking whether the patient has adopted the starting position or not properly, and on the other hand, it gets the initial values for heart rate and skin conductance. This is important, since most measurements involving these two parameters are relative, so we need a starting value. For example, not every person has same rest heart rate. Once the information from the context that each task requires has been specified, what sensor will provide it is described. In our example, the position is captured by a motion sensor, the skin conductance level is obtained by a skin conductance sensor and the heart rate is captured by a heart rate sensor. These sensors represent abstract entities, not the actual device. Therefore, now we have to specify what device will actually play the role of the abstract sensor. As depicted in Figure 8, the motion sensor role is played by a Microsoft Kinect, the heart rate sensor role is played by a Xiaomi Band S1 and, finally, the skin conductance sensor role is played by a Microsoft Band. Lastly, Figure 8 also specifies the actual context model that will handle the relevant context information. In this case, there is a *User* *ContextElement* to represent the patient. This *ContextElement* has two capabilities: *ArmsPosition* and *VitalSigns*. In *ArmsPosition* there is a *DynamicContextData* element for each part of the body whose position is relevant for the task, that is, the wrist, the elbow and the shoulder of both arms. In the same way, *VitalSigns* capability comprises those biological signals considered relevant for the exercise execution, including one *DynamicContextData* for the skin conductance and another for the heart rate.

**Figure 9:**
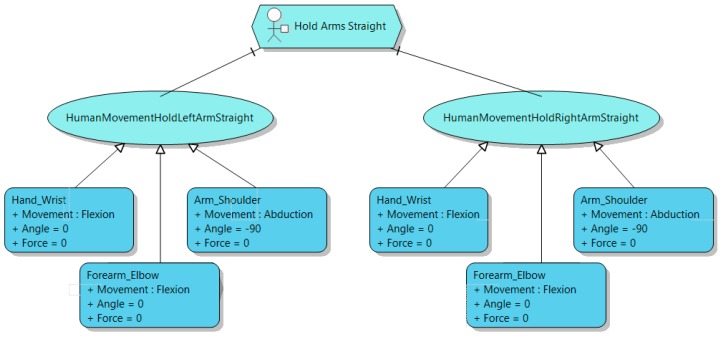
The upper limb rehabilitation presentation model.

Continuing with the specification of *Start URL* task, and once we have already described what context elements are involved and what devices will be used to get the required information from the context, now we focus on detailing accurately the movements required to achieve the task (see Figure 9).

*Start URL* is composed of two tasks, namely, *Good Seated Posture* and *Hold Arms Straight*. In this case, only *Hold Arms Straight* has been assigned a presentation, since the specialist assumes the patient knows how to get a good seated posture. *Hold Arms Straight* is also composed of two *HumanMovement* elements: hold straight left arm (*HumanMovementHoldLeftArmStraight*) and hold right arm straight (*HumanMovementHoldRightArmStraight*). Finally, each *HumanMovement* of the arm is described, in this case, by specifying the position of the arm, the forearm and the wrist. For each part of the arm an *IndividualMovement* is used to precisely describe the position the patient is asked to reach. For the *Arm_Shoulder* of both arms an abduction with an angle of −90° and no force is required. Then, for *Forearm_Elbow* of both arms, a flexion with an angle of 0° and no force is required. Lastly, for both *Hand_Wrist,* a flexion with an angle of 0° and no force is required too. Please, note the angle of a segment, such as the forearm, is relative to the previous one (the arm in our example), always starting with the closest one to the trunk. If no movement is specified for the previous segment, then the default position is assumed. Thus, the final position the user is supposed to reach is fully specified. Finally, the specialist can decide the level of tolerance, that is, how close to the ideal position the patient should reach for the position to be successful.

## 5. Conclusions and Future Work

Currently, physical rehabilitation systems aim at bridging the gap between users and technology in order to make the most of the rehabilitation process. In this sense, the development of Post-WIMP tele-rehabilitation systems, going beyond the traditional Windows, Icons, Menus and Pointer interfaces are becoming a meaningful step forward, as they facilitate the interaction between users and technology in a more natural and effective way [23]. Moreover, the exploitation of the context-aware approach enables these systems to react to the information available of users, platform and environment adapting the rehabilitation process as needed. In this paper, we have presented some of the most important meta-models that have been distilled from the development of Vi-SMARt, a context-aware Post-WIMP rehabilitation system in which we have been working during the last years. These meta-models are part of our model-based proposal for developing context-aware Post-WIMP rehabilitation systems.

This proposal encompasses three models, namely task, context and presentation models. The Task Model presented in this work has been defined to provide expressive power enough to specify contextualized and physical tele-rehabilitation tasks, that is, to specify tasks that users carry out interacting with the system. Our proposal overcomes one of the limitations of the model-based approach for the development of context-aware systems identified in [18] as this task model supports the specification of non-functional requirements. Moreover, as these tasks can be contextualized, they can be performed or monitored differently depending on the context, that is, depending on the user, platform and environment they are performed in. For this aim, the presented Context Model offers facilities to define different contexts of use that can be related to the tasks previously described. Unlike most context modeling approaches [39] we keep a separation of concerns between task and context models. Thus, those models are easier to design and understand. Furthermore, the context model devised also supports the specification of both static and dynamic data, and the different interaction modalities that a device supports. Additionally, the tasks can be enriched with awareness resources that provide extra information that contributes to improve task execution. Finally, as this work promotes the exploitation of Post-WIMP technology to provide support for the monitoring of human movements during a physical rehabilitation activity, a bio-inspired Presentation Model has been also introduced as part of our Model-Based proposal. Basically, by specifying this Presentation Model specialists are able to define exactly how patients should carry out each movement by describing the starting and final positions of the relevant body parts involved. The use of different models, for both the tasks the patient should do and how these tasks are actually accomplished, pursues a proper separation of concerns between the tasks and its presentation so that each rehabilitation activity can be customized according to the context of use and the user by choosing different suitable presentations. 

Several challenges constitute our on-going work. First, we are currently working on a tooling framework that provides code-generation facilities from the models defined. In this sense, the development of Vi-SMARt provided us with the foundations to validate the expected results. The main goal is to provide developers with a suitable framework that enables them to develop this type of application in an easy an intuitive manner. This framework will exploit he traceability of the designs created with the proposal presented in this paper to the implementation in terms of a multi-agent system [8]. Moreover, in order to achieve such goal, we are also designing some empirical experiments to evaluate, relying on developers as experimental subjects, the applicability and understandability of our proposal. These experiments will focus on the understandability of the artifacts produced while applying our approach. Furthermore, another future work also related to the evaluation of our proposal is the design of a comparative multiple-case study to find out whether our approach helps to improve the quality of the systems developed and to speed up the development process. 

## Abbreviations

The following abbreviations are used in this manuscript:

CTT	ConcurTaskTrees
CSRML	Collaborative Systems Requirements Modeling Language
GUI	Graphical User Interface
HAMSTERS	Human-centered Assessment and Modeling to Support Task Engineering for Resilient Systems
HCI	Human Computer Interaction
HTA	Hierarchical task analysis
ICT	Information and Communication Technologies
MDA	Model-Driven Architecture
MDD	Model-Driven Development
UI	User Interface
USIXML	USer Interface eXtensible Markup Language
WIMP	Window, Icon, Menu, Pointer
